# Bridging the Implementation Gap in Medical AI Education: 3-Lever Framework for Concurrent Reform

**DOI:** 10.2196/88778

**Published:** 2026-04-28

**Authors:** Sherrie L Kelly, Melissa Forgie, Mark C Walker

**Affiliations:** 1 Clinical Epidemiology Program, Ottawa Hospital Research Institute, 501 Smyth Road, Ottawa, ON, K1H 8L6, Canada, 1 613-761-4395; 2 Faculty of Medicine, University of Ottawa, Ottawa, ON, Canada; 3 Maternal Fetal Medicine, General Campus, Ottawa Hospital, Ottawa, ON, Canada

**Keywords:** artificial intelligence, AI, medical undergraduate education, curriculum, accreditation, health equity

## Abstract

The rapid integration of artificial intelligence (AI) into clinical practice necessitates urgent restructuring of medical education and physician assessment to ensure that future physicians are proficient and responsible users of AI tools. Despite the existence of core AI competencies, the current state of AI education in Canadian undergraduate medical education is highly inconsistent and disjointed, and available data indicate that most medical students receive minimal to no formal AI training even as they anticipate that AI will profoundly shape their future careers. National policy, specifically the Pan-Canadian AI for Health Guiding Principles, has advanced the agenda by calling for AI literacy among health professionals and emphasizing core values such as equity, robust data practices, and Indigenous-led data governance. However, these principles offer limited practical guidance on the educational and regulatory mechanisms required for effective implementation. We contend that this critical implementation deficit arises from a traditional, sequential reform model in which faculty development, curriculum change, and regulatory updates occur in isolation. This slow, siloed approach is fundamentally inadequate for addressing AI’s inherent speed, opacity, and significant equity risks. To overcome this challenge, we propose a 3-lever concurrent implementation framework that provides a conceptual lens to address the interdependencies among faculty development, curriculum, and regulation. This model posits that AI competencies transition from abstract requirements to practical application only when 3 levers—clinician-educator capacity, digitally enabled learning environments, and regulatory and assessment reform—are activated simultaneously and in alignment. This Viewpoint extends existing AI competency frameworks by theorizing AI curriculum implementation as a problem of concurrent lever activation and by outlining minimum concurrent actions for deans and regulators that can be adapted to competency-based medical education systems. Although illustrated with Canadian examples, the framework is designed to be transferable beyond Canada and offers a testable, licensure-level blueprint for embedding AI competence in medical education.

## Introduction

Artificial intelligence (AI)—machine-based systems that support diagnosis, risk stratification, workflow, or documentation—is now woven into clinical practice; however, undergraduate medical curricula reflect a pre-AI era. We hypothesize that unless 3 system levers—human capacity, learning environments, and regulation—are activated together, AI competencies will remain optional and inequitable. This framework synthesizes existing evidence and provides a theoretical foundation for addressing the AI implementation gap. While the framework is grounded in current knowledge, future research should focus on evaluating its practical application and assessing its effectiveness across different medical education systems, including institutional settings, regulatory frameworks, and levels of training.

Singla et al [1] have defined core AI learning elements for Canadian undergraduate medical education (UGME). Similarly, reviews and conceptual syntheses argue that AI and data science literacy should be core competencies, organized around domains such as ethics, application, communication, and quality improvement. Most efforts have been small elective pilots with limited system-level uptake [2-5]. Our 3-lever framework extends this foundation by explaining why familiar levers—faculty development, learning environments, and regulatory mandates—have not yet moved in concert for AI and by theorizing how their interaction can shift AI competence from an aspirational concept to an assessable requirement for licensure.

Across world regions, competency-based medical education systems face an AI implementation gap: competency frameworks exist; however, clinical training continues to lag [2,3,5]. Unlike static knowledge domains, AI’s velocity, opacity, and inherent risk of inequity render the conventional sequential activation of these levers inadequate and unsafe. Our contribution is to frame this implementation problem as a theory of concurrent lever activation. We argue that AI competence will remain optional and inequitable as long as faculty development, learning environments, and regulatory instruments move sequentially and in isolation. Existing AI competency lists and frameworks primarily define *what* learners should know about AI, whereas most digital health and AI education implementation reports focus on a single lever, for example, introducing new courses, local pilots, or technical infrastructure without concurrent regulatory change [2-5]. In contrast, generic calls for “AI literacy” often stop at high-level advocacy without specifying which system levers must change together. Therefore, our 3-lever framework is deliberately positioned as an implementation theory that complements content-focused competency lists and single-lever curricular reports by modeling how faculty, learning environments, and regulatory instruments must be activated concurrently for AI competence to become a licensure-level requirement rather than an optional enrichment.

The 3-lever framework differs from previous AI curriculum work in 2 ways. First, it explains recurring failure modes when levers move alone, for example, faculty-led initiatives that leave students experimenting unsupervised with powerful tools or regulatory reforms that assess AI knowledge abstractly without supporting practice in digital environments. Second, it generates operational prescriptions and predictions about how specific combinations of lever activation are likely to shape learner exposure, assessment stakes, and equity outcomes.

For example, if institutions activate only lever 2 (curriculum) by introducing AI electives without lever 1 (faculty development) or lever 3 (assessment), AI exposure will remain elective, champion-dependent, and vulnerable to rapid obsolescence. If regulators move only on lever 3 by adding AI items to licensure examinations without corresponding curricular and digital infrastructure, learners will memorize AI terminology without practicing AI-mediated clinical reasoning. If health systems adopt clinical AI tools without educational levers, students will first encounter AI through unsupervised clinical use rather than in safe sandboxes. Therefore, the framework functions as a predictive implementation model for AI in medical education. Specifically, it generates testable predictions that can be examined empirically, including the following: if only lever 2 (curriculum and learning environments) is activated, AI exposure will remain elective, uneven, and heavily dependent on local champions; if levers 1 and 2 move without Lever 3 (regulation and assessment), AI teaching and digital sandboxes will expand but remain low stakes and easily displaced when curricular time is constrained; if lever 3 moves without levers 1 and 2, high-stakes examinations will incentivize rote memorization of AI terminology rather than supervised practice in AI-mediated clinical reasoning; and if all 3 levers are activated concurrently, AI competence is more likely to become a licensure-level expectation with observable performance in digital clinical environments, including competencies related to equity and data governance.

**Figure 1.**
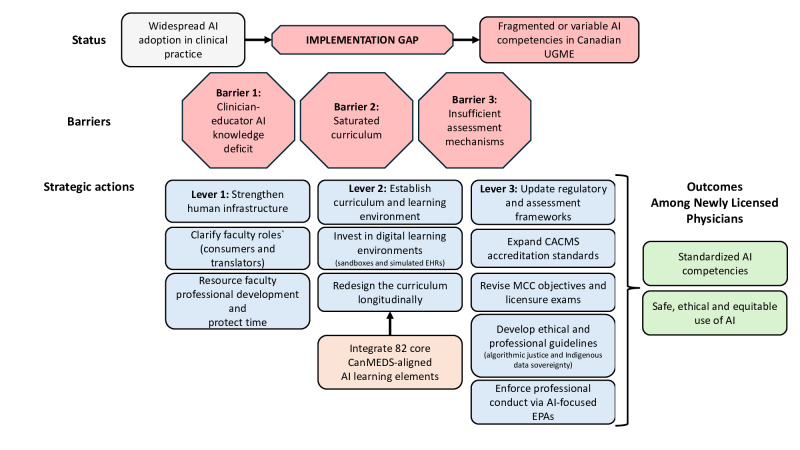
Multisystem framework for bridging the artificial intelligence (AI) competency gap in undergraduate medical education (UGME). The 82 CanMEDS (Canadian Medical Education Directives for Specialists)–aligned AI learning elements referred to in the figure were developed by Singla et al [1]. CACMS: Committee on Accreditation of Canadian Medical Schools; EHR: electronic health record; EPA: entrustable professional activity; MCC: Medical Council of Canada.

As summarized in Figure 1 [1], lever 1 strengthens human infrastructure by supporting AI- literate clinician-educators. Lever 2 redesigns the curriculum and learning environments, including digital infrastructure such as sandboxes and simulated electronic health records (EHRs). Lever 3 aligns regulatory and assessment frameworks—accreditation standards, Medical Council of Canada (MCC) objectives, licensure examinations, and entrustable professional activities (EPAs)—to make AI literacy a nonnegotiable requirement. Throughout, reforms must be operationalized through algorithmic justice and Indigenous data sovereignty—not as add-ons but as primary lenses for safety [6-8]. Although we focus on Canadian UGME, similar competency- based systems internationally face parallel challenges, and our framework is applicable across the continuum, including in postgraduate medical education and continuing professional development (postgraduate medical education alignment is illustrated in Table 1). It can also be adapted for decentralized systems, such as those in Europe, by accounting for regional variability in regulatory structures and educational priorities while maintaining its core principles.

**Table 1.** Regulatory and assessment alignment for artificial intelligence (AI) competence across the Canadian and US undergraduate medical education (UGME)–postgraduate medical education (PGME) continuum.

Phase	Core AI competency focus	Lever 1: faculty capacity (who)	Lever 2: learning environment (how)	Lever 3: regulation and assessment (what)
UGME (foundation)	AI literacy, safety, and ethics with focus on understanding bias, data provenance, and critical appraisal of AI-generated information	Designate clinical AI leads for curriculum mapping and longitudinal faculty mentoring	Integrate AI training longitudinally into core clerkship and ethics sessions and practice in shared digital sandboxes	Canadian alignment: Committee on Accreditation of Canadian Medical Schools standards and Medical Council of Canada objectives (digital health focus) US alignment: Liaison Committee on Medical Education standards and United States Medical Licensing Examination step 1 or 2
PGME (application)	Safe, supervised AI, and oversight with focus on specialty-specific applications, identifying system failures, and QI^a^ related to AI	Embedding of AI competency into academic half-day and QI projects with dedication of AI-focused faculty mentors in subspecialties	Use of AI-enabled electronic health records in workplace-based assessment, with mandatory debriefs on automation bias cases	Canadian alignment: Royal College of Physicians and Surgeons of Canada or College of Family Physicians of Canada EPAs^b^ US alignment: Accreditation Council for Graduate Medical Education milestones and specialty-specific EPAs

a QI: quality improvement.

b EPA: entrustable professional activity.

## Policy to Practice

Our Viewpoint draws on 4 strands of literature and policy. First, the expert-based curriculum developed by Singla et al [1] provides detailed CanMEDS (Canadian Medical Education Directives for Specialists)–aligned AI learning objectives for UGME, specifying what students should know. However, it is largely agnostic about system- level levers—accreditation, licensure, and national assessment—that would make these competencies mandatory.

Second, recent reviews and a conceptual paper argue that AI and data science literacy should be core training outcomes and find that most AI curricula remain fragmented and elective [2-5]. Few are anchored in high- stakes assessment or accreditation, leaving exposure variable and dependent on local champions.

Third, empirical studies of Canadian learners confirm this patchwork reality. A mixed methods study across Canadian medical schools found that most students (85%) reported no formal AI education despite anticipating that AI would significantly affect their future practice [9]. A national survey of health professional students found similar patterns: most predicted AI would shape their careers yet felt unprepared and unsure how to respond [10].

Fourth, the AI for Health (AI4H) Guiding Principles, endorsed by Canadian health ministers, set a policy context that stresses AI literacy, equity, and Indigenous- led data governance and calls for robust oversight across health systems [6]. However, AI4H does not articulate specific educational reforms or regulatory mechanisms, instead assuming that health professionals will be able to understand, critique, and oversee AI systems without specifying how or where that competence will be cultivated and verified.

Taken together, these strands show that Canada has detailed AI competencies for UGME, evidence that learners are not consistently achieving them, and a policy framework that demands AI- literate, equity-focused physicians—but no coherent implementation strategy linking competencies, curricula, faculty development, infrastructure, accreditation, and assessment. Existing work has described what to teach and how AI should behave but lacks a theory explaining why AI competencies have not become routine and which system levers must move together to change that. While this framework addresses these gaps, its successful implementation will require careful navigation of political and economic barriers, including stakeholder alignment, resource allocation, and competing institutional priorities. The 3-lever framework provides such a theory by linking content frameworks (eg, the curriculum developed by Singla et al [1]), learner surveys, and policy principles (eg, AI4H) to specific combinations of faculty development, learning environments, and regulatory instruments and by making testable predictions about how different patterns of lever activation will entrench or narrow the current implementation gap.

In this Viewpoint, we synthesize national policy, competency frameworks, and empirical work with Canadian learners to propose a 3-lever concurrent implementation model for AI in UGME. We show how different patterns of lever activation predict whether AI competence remains optional or becomes a licensure-level expectation and outline concrete regulatory and curricular actions that can be adapted to competency-based medical education systems internationally.

## Lever 1: Human Infrastructure

Lever 1 focuses on who can teach and supervise responsible AI use: the human infrastructure of clinician-educators. Most clinicians were trained in a pre-AI era and report limited confidence in teaching, supervising, or evaluating AI-mediated clinical reasoning [11].

Faculty development in clinical AI should be treated as foundational infrastructure rather than optional enrichment, given its necessity for safe clinical oversight. This includes equipping educators with key competencies such as critically appraising AI models, interpreting performance metrics, identifying biases, and supervising learners in the use of AI tools within clinical contexts. Without AI-literate educators, even well-designed curricula will be delivered inconsistently, and learners will likely rely on unsupervised experimentation with powerful tools. Development efforts should be longitudinal and rapid-cycle, focusing less on turning clinicians into data scientists and more on oversight skills that they already apply to other technologies: understanding how AI models are developed and validated, interpreting performance metrics, recognizing when models are used outside their intended population or context, and supervising learners who use AI- enabled tools [2-5].

Resourcing faculty development is essential. Without protected time and institutional recognition, programs risk becoming perfunctory. Institutions should leverage funding for teaching innovation; allocate dedicated time to clinical AI leads; and recognize AI-focused teaching, mentoring, and curriculum reform in promotion and merit processes. While some Canadian faculties have begun appointing clinicians with expertise in AI and education as “clinical AI leads,” this role remains aspirational in many institutions and requires support to become widespread. These roles typically focus on faculty mentoring, curriculum design, and serving as liaisons with data governance structures.

Operationally, the 3-lever framework implies that schools should treat AI-focused faculty development as minimum safety infrastructure rather than optional enrichment. At a minimum, each school should designate a clinical AI lead with protected time, embed AI oversight skills into existing faculty development programs for clinical teachers, and track whether every clerkship discipline has at least 1 educator capable of supervising AI-mediated clinical reasoning.

A common counterargument is that increasingly user- friendly AI interfaces will allow clinicians to use them safely without extensive training [12]. This view underestimates both the sociotechnical complexity of clinical AI and the evidence from safety science. Studies on automation bias—the tendency to overtrust automated recommendations—show persistent risk of overreliance on algorithms, underrecognition of new forms of error, and diffusion of responsibility when decision support systems are introduced [7,12,13]. A study on AI- driven decision-making similarly cautions that apparent fluency can mask shallow understanding [14]. Without faculty who can teach these hazards and model algorithmic skepticism—a stance of critical engagement with AI outputs that prioritizes identifying and mitigating bias and equity harms—AI tools may be used uncritically [7,12].

## Lever 2: Curriculum and Learning Environments

Lever 2 focuses on where and how learners encounter AI: curricula and learning environments. Singla et al [1] recommend that AI learning be woven longitudinally throughout UGME rather than confined to stand- alone electives, a view echoed by global reviews that find that piecemeal, elective-based approaches reinforce inequities in exposure and rarely shape assessment or accreditation [2-5].

Early in training, students can be introduced to core AI and data science concepts—such as how bias enters through data collection, labeling, and outcome selection—through case- based discussions and clinical examples. These cases must be designed to interrogate the ethical and sovereignty implications of data used for training AI systems [6,8]. Later, as students move into clinical skills courses and clerkship, AI-enabled tools should appear in realistic scenarios, for example, decision support suggesting a differential diagnosis or a risk score influencing triage. Cases should require learners to reconcile AI outputs with examination findings, patient narratives, and contextual information so that AI becomes part of routine clinical reasoning pedagogy rather than an isolated topic.

Generative AI (GenAI) further adds complexity. Large language models now perform at or near passing thresholds on United States Medical Licensing Examination (USMLE)– style assessments, raising questions about what such tests measure [14,15]. Curricula should address GenAI both as content and as context: students should learn responsible use (disclosure, verification, and professional boundaries), and assessment formats that are easily outsourced to GenAI (eg, unsupervised take- home essays) will require redesign to reflect individual competence and uphold academic integrity [16,17]. This connects lever 2 to lever 3 because GenAI forces reconsideration of both teaching strategies and assessment design.

## Digital Learning Environments

Even a well-designed curriculum will fall short if learners never interact meaningfully with AI tools. We suggest that faculties invest in secure digital “sandboxes” and simulated EHRs that embed AI decision support tools. In a sandbox, students can explore how models behave with different inputs, observe performance changes when data distributions shift, and experiment with prompting strategies for GenAI using deidentified or synthetic data in a test environment [10]. This allows observation rather than merely reading about phenomena such as model drift, bias, and overfitting. For example, a second-year student might work through a simulated emergency department case in which entering different vital signs, comorbidities, and social history variables changes an AI-generated sepsis risk score, and the student would then be required to document why they accept or override the recommendation before debriefing with a faculty supervisor. Such concrete exercises make abstract concepts such as distribution shift, calibration, and automation bias visible and discussable.

Simulated EHRs can extend this experience to realistic workflows. Learners can navigate simulated electronic charts in which AI- driven tools surface alerts, risk scores, or suggested orders, practicing when to accept, override, or ignore AI outputs and how to explain those decisions to patients and colleagues.

From a 3-lever perspective, digital sandboxes and simulated EHRs are not aspirational extras but mechanisms that make AI competence observable. They translate abstract competencies into tasks that can be practiced and assessed, such as overriding an unsafe AI recommendation or explaining an AI-generated risk score to a patient. Without these environments, curricular objectives remain largely theoretical and cannot meaningfully support the regulatory changes proposed under lever 3.

Building this infrastructure is resource intensive. Relying solely on local resources risks creating a digital divide between medical schools. We propose leveraging existing national digital health partnerships to coordinate shared, cloud-based sandboxes. The long-term cost of graduating physicians who learn AI through unsupervised, unsafe experimentation on patients is likely higher than the investment in shared training environments. Shared infrastructure ensures equitable access without duplicating costs at every institution. We recognize the constraints facing underresourced schools; therefore, a balanced implementation must also support agile, low-resource curricular integration models in parallel with national infrastructure development.

## Lever 3: Regulatory and Assessment Frameworks

Lever 3 focuses on what makes AI competence nonoptional: the regulatory and assessment frameworks that shape institutional behavior. Modernizing faculty development and curricula will have limited impact if AI competence remains optional from the standpoint of accreditation and licensure. Coordinating action across accrediting bodies, licensing boards, and Indigenous communities presents significant challenges, including jurisdictional variability, misaligned incentives, and resistance to change. However, these barriers can be addressed through clear mandates from national bodies, alignment of incentives (eg, funding for compliance), and inclusive governance structures that prioritize equity and Indigenous leadership.

As seen in other areas such as patient safety and quality improvement, accreditation standards, national objectives, licensure examinations, and EPAs have historically driven change in Canada. For example, when national bodies strengthened patient safety and quality improvement expectations within accreditation standards and objective structured clinical examination (OSCE) blueprints, many schools rapidly introduced new safety curricula, checklists, and simulation cases to avoid accreditation and examination risk; AI-related objectives are likely to exert similar system-level effects once they are explicitly blueprinted into high-stakes assessments. AI literacy, including its equity and data governance dimensions, will likely need to follow a similar path. Although we illustrate lever 3 using Canadian (Canadian Accreditation Council for Medical Schools and MCC) and US (Liaison Committee on Medical Education and USMLE) structures, the underlying principle—making AI competence a licensure-level expectation rather than an optional elective—applies to any competency-based medical education system.

At present, AI-related competencies are largely implicit or absent from these frameworks. Accreditation standards from the Committee on Accreditation of Canadian Medical Schools and postgraduate bodies emphasize broad domains such as patient safety, professionalism, and use of health information technologies, which are relevant to AI but do not explicitly mandate AI competencies. We suggest that accrediting bodies articulate expectations that graduates can interpret and critically appraise AI tools, understand their equity and data sovereignty implications, and function safely in AI- enabled clinical environments [18]. Standards should focus on outcomes and principles rather than prescribing detailed curricula, for example, requiring evidence of a longitudinal AI learning thread mapped to national competencies, AI- related faculty development, and explicit attention to AI4H principles and Indigenous data sovereignty within ethics and professionalism curricula while leaving design to local context [1,4,6].

Licensure and certification examinations are equally important. The MCC objectives (revised 2022) explicitly address general competence in digital health, including AI and machine learning, but a detailed assessment framework is still needed. As the Council is in the process of modernizing its digital infrastructure, it has an opportunity to incorporate AI- related content into examination objectives and instruments [19]. We suggest that the MCC objectives be revised so that graduates are expected to interpret AI outputs and performance metrics in a clinical context, recognize bias and equity implications, and communicate AI-related benefits and limitations to patients. These expectations should be assessed through written questions that require candidates to critique AI- generated outputs or identify unsafe uses and through OSCE stations in which candidates navigate conflicts between AI recommendations and clinical judgment or obtain informed consent for AI- mediated care [7,14]. EPAs can then embed AI competence into workplace- based assessment by translating broad competencies into observable units of work [20].

Concretely, the framework predicts that once AI-related objectives are tied to blueprinted examination content and OSCE stations that explicitly require candidates to critique AI outputs, schools will reallocate curricular time and faculty development resources toward AI competence to avoid accreditation and licensure risk. Conversely, if AI remains absent from high-stakes assessment, even well-designed curricula and sandboxes will remain vulnerable to marginalization when time and resources are constrained.

AI evolves faster than assessment cycles, so examining bodies are strongly encouraged to adopt agile, rapid-cycle blueprint reviews. This challenge is not unique to Canada. The 3-lever framework applies to any competency-based system; for example, aligning lever 3 with USMLE content outlines would drive lever 2 resource allocation in US medical schools. Aligning AI competencies with existing high-stakes regulatory instruments—whether the MCC, the Liaison Committee on Medical Education, or the Accreditation Council for Graduate Medical Education—is more effective than creating entirely new governance structures.

## Ethics and Indigenous Data Sovereignty

Technical literacy alone is insufficient for safe clinical AI. Much of the early AI education literature emphasizes how models work and how they might improve efficiency or accuracy [2,3,5], with less attention given to how AI systems can reproduce inequities or to the rights of Indigenous Peoples over data that concern them. In Canada, AI4H explicitly names equity, Indigenous data sovereignty, and frameworks such as OCAP (ownership, control, access, and possession) and the CARE (collective benefit, authority to control, responsibility, and ethics) principles as core requirements for health-related AI [6,8].

We propose organizing ethical and professional teaching about AI around 2 interlocking concepts: algorithmic justice and Indigenous data sovereignty. Algorithmic justice focuses on how seemingly neutral technical decisions—such as which outcomes to predict, what data sources to include, or which proxy variables to use—can embed historical and structural inequities into AI systems [10]. For example, students can examine how pulse oximetry, dermatology image datasets underpinning diagnostic algorithms, or historical estimated glomerular filtration rate equations have systematically misclassified risk in racialized populations and can practice explaining these failures to patients and interprofessional teams. Students should interrogate AI case studies through an equity lens, asking: Who is represented in the training data? Which populations are missing? How does performance vary across groups? What types of harms might fall on those least represented in the training data? [6,7] These exercises move discussions of fairness from abstract principles to concrete professional responsibilities and should be integrated into clinical reasoning, ethics, and quality improvement components of the curriculum, rather than confined to stand-alone lectures.

Indigenous data sovereignty extends these questions by asserting that Indigenous nations have the right to govern the collection, ownership, use, and sharing of data about their peoples, lands, and knowledge [8]. In a Canadian context, this includes instances in which administrative or research datasets have been used to develop AI models about Indigenous communities without respecting community-defined governance processes or data sharing agreements. This is directly relevant to AI systems trained on health, genomic, or geospatial data that include Indigenous communities. Canadian guidance emphasizes that Indigenous data governance frameworks should inform health system practices and that AI principles must respect Indigenous governance [6,8]. Therefore, UGME should include Indigenous- led teaching on data sovereignty and AI, co-designed with Indigenous organizations and aligned with reconciliation commitments, and graduates should be able to recognize when a proposed AI deployment lacks explicit Indigenous data governance agreements and escalate such concerns appropriately.

Algorithmic justice and Indigenous data sovereignty are central to all 3 levers. Faculty development (lever 1) should equip educators to teach about bias, inequity, and data governance. Curricular design and digital environments (lever 2) should embed simulations that surface these issues, rather than presenting only “average” patients. Regulatory and assessment frameworks (lever 3) should explicitly reference equity and data sovereignty expectations, so that graduates are assessed not only on technical understanding but also on ethical and community-responsive use of AI. Concretely, programs can blueprint at least 1 written examination question and 1 OSCE or simulated case in which learners must recognize and articulate an algorithmic bias or a breach of an Indigenous data governance agreement and propose an alternative course of action. Assessing these scenarios signals that graduation and licensure depend not only on technical understanding but also on ethical and community-responsive use of AI.

## Conclusions

Frameworks such as the 82 essential AI competencies for Canadian UGME [1] and policy documents such as AI4H [6] set expectations for literacy, safety, equity, and Indigenous data sovereignty. The central question for academic medicine is how to move from these frameworks and principles to practice so that every graduating physician can use AI responsibly.

We have argued that closing this implementation gap requires coordinated action across 3 levers—human infrastructure, curriculum and learning environments, and regulatory systems. Textbox 1 and Table 1 translate this framework into a set of minimum concurrent actions and illustrative alignments across training phases and jurisdictions. This framework builds on existing evidence and offers a structured approach to embedding AI competencies into medical education. While grounded in evidence, its implementation across diverse settings will offer opportunities for refinement and optimization.

AI’s urgency and interconnectedness favor applying these 3 levers concurrently rather than sequentially. However, their application must account for the complexity of nested systems with competing priorities, requiring adaptive and context-sensitive approaches. Faculties should invest in AI-literate clinician-educators; redesign curricula to integrate AI into clinical reasoning, ethics, and systems- based practice, taught in partnership with Indigenous communities and equity experts and practiced within digital sandboxes and simulated EHRs [1,4,5]; and collaborate with accrediting bodies, the MCC, and specialty colleges to embed AI competencies into accreditation standards, examination objectives, and EPAs, with mechanisms for rapid-cycle adjustment.

Textbox 1. **Minimum concurrent actions for deans and regulators to embed artificial intelligence (AI) competence in undergraduate medical examination.**

**Lever 1: human infrastructure**

- Appoint at least 1 clinical AI lead or a small team (eg, 0.1‐0.2 full-time equivalent each) at every medical school to coordinate AI teaching and supervision
- Integrate a short AI oversight module (eg, 3‐4 sessions) into existing faculty development programs, focusing on supervising AI-mediated clinical reasoning
- Ensure, at the program level, that each core clerkship has access to at least 1 AI-literate supervisor for learners

**Lever 2: curriculum and learning environment**

- Map the 82 CanMEDS (Canadian Medical Education Directives for Specialists)–aligned AI learning elements onto existing courses and clinical experiences to identify overlaps and gaps
- Introduce at least 1 shared digital sandbox or simulated electronic health record exercise in both preclerkship and clerkship phases in which students must work with AI outputs
- Embed longitudinal case discussions in which students reconcile AI recommendations with physical examination findings, social history, and patient preferences

**Lever 3: regulatory and assessment frameworks**

- Add explicit AI-related enabling language to accreditation self-study templates and program evaluation reports
- Blueprint a defined proportion (eg, 5%‐10%) of written examination content and objective structured clinical examination stations for AI literacy, equity, and data governance objectives
- Develop or adapt at least 1 entrustable professional activity that requires safe, supervised use of AI tools in clinical decision-making as a condition of graduation or licensure

Future work should evaluate how different models of faculty development, simulation design, and regulatory reform influence AI oversight skills, susceptibility to automation bias, and downstream outcomes such as quality of care and equity and should examine how Indigenous-led data governance frameworks can be authentically incorporated into medical education and how these levers can be adapted for diverse resource settings and regulatory architectures [6,8]. If Canadian medical education fails to act, physicians risk graduating underprepared for AI-mediated practice, with consequences for patient safety, equity, and public trust. Conversely, timely and coordinated reform—grounded in algorithmic justice and Indigenous data sovereignty and operationalized through the 3 levers we propose—can help ensure that AI becomes a well-governed tool that supports safer, more ethical, and more equitable care across Canada and beyond.

## Abbreviations

AI: artificial intelligence
AI4H: Artificial Intelligence for Health
CanMEDS: Canadian Medical Education Directives for Specialists
CARE: collective benefit, authority to control, responsibility, and ethics
EHR: electronic health record
EPA: entrustable professional activity
GenAI: generative artificial intelligence
MCC: Medical Council of Canada
OCAP: ownership, control, access, and possession
OSCE: objective structured clinical examination
UGME: undergraduate medical education
USMLE: United States Medical Licensing Examination
